# Evaluating parents’ decisions about next-generation sequencing for their child in the NC NEXUS (North Carolina Newborn Exome Sequencing for Universal Screening) study: a randomized controlled trial protocol

**DOI:** 10.1186/s13063-018-2686-4

**Published:** 2018-06-28

**Authors:** Laura V. Milko, Christine Rini, Megan A. Lewis, Rita M. Butterfield, Feng-Chang Lin, Ryan S. Paquin, Bradford C. Powell, Myra I. Roche, Katherine J. Souris, Donald B. Bailey, Jonathan S. Berg, Cynthia M. Powell

**Affiliations:** 1 Department of Genetics, University of North Carolina at Chapel Hill, Chapel Hill, NC 27599 USA; 2 Department of Biomedical Research, Hackensack University Medical Center, Hackensack, NJ 07601 USA; 3 Center for Communication Science, RTI International, Research Triangle Park, NC 27709 USA; 4 Department of Pediatrics, University of North Carolina at Chapel Hill School of Medicine, Chapel Hill, NC 27599 USA; 5 Department of Biostatistics, University of North Carolina at Chapel Hill, Chapel Hill, NC 27599 USA; 6 Department of Heath Behavior, Gillings School of Global Public Health, University of North Carolina at Chapel Hill, Chapel Hill, NC 27599 USA; 7 Center for Newborn Screening, Ethics, and Disability Studies, RTI International, Research Triangle Park, NC 27709 USA

**Keywords:** Translational genomics, Sequencing, Newborn screening, Parental decision aid, Randomized trial

## Abstract

**Background:**

Using next-generation sequencing (NGS) in newborn screening (NBS) could expand the number of genetic conditions detected pre-symptomatically, simultaneously challenging current precedents, raising ethical concerns, and extending the role of parental decision-making in NBS. The NC NEXUS (Newborn Exome Sequencing for Universal Screening) study seeks to assess the technical possibilities and limitations of NGS-NBS, devise and evaluate a framework to convey various types of genetic information, and develop best practices for incorporating NGS-NBS into clinical care. The study is enrolling both a healthy cohort and a cohort diagnosed with known disorders identified through recent routine NBS. It uses a novel age-based metric to categorize a priori the large amount of data generated by NGS-NBS and interactive online decision aids to guide parental decision-making. Primary outcomes include: (1) assessment of NGS-NBS sensitivity, (2) decision regret, and (3) parental decision-making about NGS-NBS, and, for parents randomized to have the option of requesting them, additional findings (diagnosed and healthy cohorts). Secondary outcomes assess parents’ reactions to the study and to decision-making.

**Methods/design:**

Participants are parents and children in a well-child cohort recruited from a prenatal clinic and a diagnosed cohort recruited from pediatric clinics that treat children with disorders diagnosed through traditional NBS (goal of 200 children in each cohort). In phase 1, all parent participants use an online decision aid to decide whether to accept NGS-NBS for their child and provide consent for NGS-NBS. In phase 2, parents who consent to NGS-NBS are randomized to a decision arm or control arm (2:1 allocation) and learn their child’s NGS-NBS results, which include conditions from standard (non-NGS) NBS plus other highly actionable childhood-onset conditions. Parents in the decision arm use a second decision aid to make decisions about additional results from their child’s sequencing. In phase 3, decision arm participants learn additional results they have requested. Online questionnaires are administered at up to five time points.

**Discussion:**

NC NEXUS will use a rigorous interdisciplinary approach designed to collect rich data to inform policy, practice, and future research.

**Trial registration:**

clinicaltrials.gov, NCT02826694. Registered on 11 July, 2016.

**Electronic supplementary material:**

The online version of this article (10.1186/s13063-018-2686-4) contains supplementary material, which is available to authorized users.

## Background

Universal newborn screening (NBS) is a highly successful public health program through which early detection and effective intervention result in documented benefits. Most countries have established criteria for which disorders to include in NBS. The United States, for example, is guided by the Recommended Uniform Screening Panel (RUSP), a list of conditions that meet strict evidence-based standards for demonstrated benefits [1]. More variability is evident in the secondary conditions that are reported, for disorders that do not meet the primary criteria for screening but nonetheless are detected [1, 2]. Advances in technology, such as next-generation sequencing (NGS), could greatly expand the number of conditions detected by NBS. The potential benefits of expansion are, however, tempered by the much slower rate of the development of effective treatments for the large number of conditions that could potentially be identified. The gap between enhanced diagnostic ability and the lack of effective treatments means that many identified conditions would not meet the standard criteria for inclusion in NBS. In addition, screening healthy newborns using NGS will create novel and profound implementation challenges and pose significant ethical considerations that may conflict with current policies governing the role of parental consent for testing. A rigorous scientific and ethical examination of the utility, acceptability, and consequences of using NGS for NBS is essential before widespread implementation can be considered [3–8].

Current applications of NGS targeted to newborns and children include its use in the diagnosis of critically ill infants with suspected genetic disorders [1, 6, 9–11]; however, it is the potential application of NGS to screen healthy newborns that creates novel challenges. The U.S. Department of Health and Human Services Secretary’s Advisory Committee on Heritable Disorders in Newborns and Children conducts a rigorous condition-by-condition review as the basis for RUSP [2]. The introduction of NGS as a screening modality would alter the current assessment of the risks and benefits of NBS because it can identify conditions for which early identification and treatment do not confer strictly defined medical benefits as well as those conditions that lack any established medical treatments [12, 13]. As a result, there is great interest in considering how meaningful parental informed consent about a range of conditions for which their newborn could be tested might be incorporated into NBS—a change that will require a deeper understanding of how parents might make these decisions [14–17].

The introduction of NGS to NBS would also challenge the precedent of confining NBS to detecting childhood-onset conditions that require effective treatment to be initiated soon after diagnosis [2, 3, 7]. Currently, the onset of symptoms of some NBS conditions, such as medium chain acylCoA-dehydrogenase deficiency (MCADD), may be delayed into adulthood due to variable expressivity; however, NGS could deliberately target adult-onset conditions, evoking important questions about which age standards are appropriate for an NBS policy. Likewise, the universal disclosure of carrier status for recessive conditions, which usually have few or no immediate health implications for the individual but may inform later reproductive decisions, would conflict with current ethical guidelines regarding pediatric genetic testing [3, 18] and (since most individuals will have several findings indicating carrier status) effectively turn the public health NBS program into a massive carrier screening program.

Some advocates have urged that the concept of direct benefits is expanded to include other relatives, such as parents, since in many cases of dominant adult-onset conditions, the parents could unknowingly have the same pathogenic variant as their child, thus potentially shortening their lifespan and subsequently impacting the child’s well-being [6, 19]. In such cases, parents would be making a decision about learning results that could directly benefit themselves at the cost of negating the child’s capacity to make this decision in the future. Perhaps equally contentious is the possibility that genomic sequencing could identify variants that predict a childhood-onset condition for which little or no effective treatment currently exists. As a result, defining the criteria for determining which information should be sought and disclosed to parents could be one of the thorniest implementation challenges facing clinicians and policy makers. Asking parents to make decisions about the types of NBS results they wish to learn would fundamentally alter the role parents have traditionally played in this setting and would create an urgent need to develop effective decision-support tools.

The NC NEXUS (North Carolina Newborn Exome Sequencing for Universal Screening) study is designed to address some of these issues by evaluating how the use of NGS can extend the utility of current NBS. One important feature of the study is that it recruits two cohorts of children: one cohort with a condition recently diagnosed through standard NBS and one cohort of newborns identified during a healthy pregnancy (see below). Recruiting diagnosed children enables a novel evaluation of the sensitivity of NGS-NBS, ascertained by comparing NGS-NBS results to genes associated with the diagnosed children’s underlying condition (diagnosed cohort only). A second important feature of the study is an embedded two-arm parallel-group randomized control trial. After learning their child’s NGS-NBS results, families will be randomized either to have or not to have the choice to learn additional findings from their child’s sequencing. This study design will allow us to investigate differences in decision regret in the two arms of the study, which is the second primary outcome of the study. The third primary outcome is parents’ decisions about NGS-NBS and, for those randomized to be able to learn them, additional findings. We will also investigate secondary outcomes that assess parents’ reactions to the study and to decision-making. This paper summarizes the NC NEXUS protocol, which is designed to achieve the foregoing aims.

## Methods/design

### Study design overview

NC NEXUS is one of four projects run by the Newborn Sequencing in Genomic Medicine and Public Health (NSIGHT) consortium, jointly funded by the Eunice Kennedy Shriver National Institute for Child Health and Human Development and the National Human Genome Research Institute. These studies explore, in a limited but deliberate manner, the implications, challenges, and opportunities associated with the possible use of genomic sequence information in the newborn period [11]. Each NSIGHT project acquires and analyzes genomic datasets that considerably expand the scale of data that has historically been available for analysis in the newborn period.

**Fig. 1:**
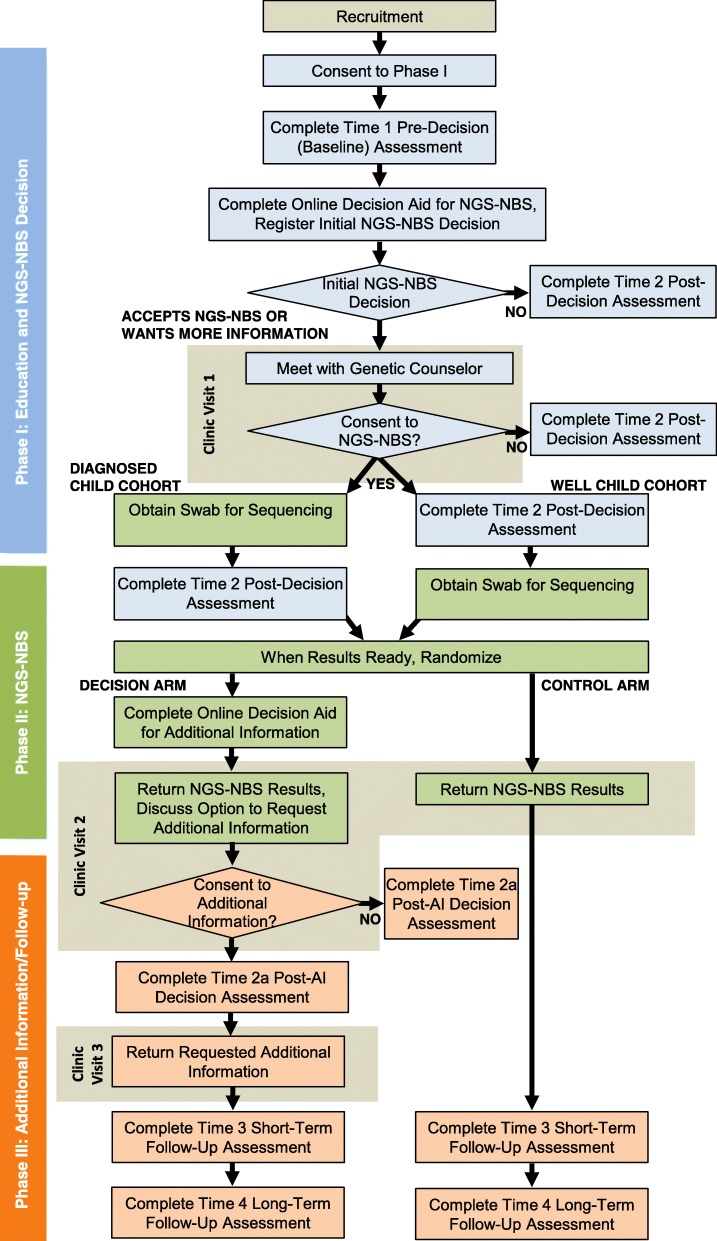
Study design workflow. Three phases of the NC NEXUS study workflow. During phase 1, interested parents agree to learn more about the study and are asked to complete the time 1 pre-decision questionnaire and the time 2 post-decision assessment. Parents use the decision aid to learn about the study and NGS-NBS for their child and complete informed consent procedures at a clinic study visit if they decide to participate. During phase 2, samples are obtained after consent is given at study visit 1. Randomization status and NGS-NBS results are returned to parents at their second study visit. During phase 3, parents in the decision arm decide via a second part of the decision aid whether to consent to and receive any additional information by phone (for carrier status results) or at a third study visit. Parents randomized to the decision arm also complete a time 2A post-additional information decision assessment. Both the decision arm and the control arm complete a short-term follow-up assessment and a long-term follow-up assessment. NBS newborn screening, NGS next-generation sequencing

**Fig. 2:**
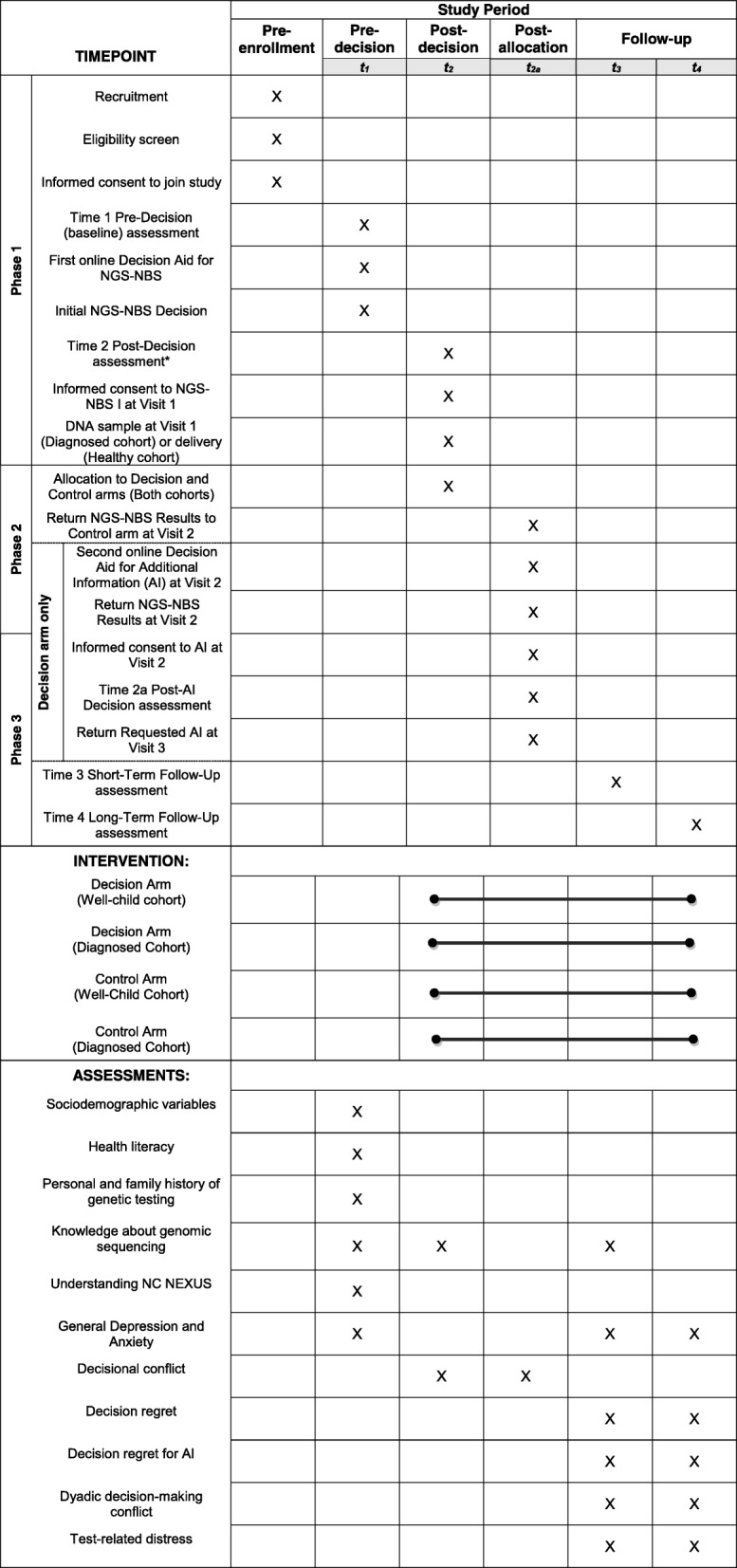
SPIRIT figure. Schedule of enrolment, interventions, and assessments. AI additional information, NBS newborn screening, NGS next-generation sequencing

The NC NEXUS project studies parental decision-making about accepting genomic information about medically actionable disorders of childhood for their child or infant using a tiered informed consent process and a two-arm parallel-group randomized controlled trial conducted in a large U.S. academic medical center (Additional file 1). In addition, after learning those results, parents who are randomized to a decision arm are asked whether they wish to learn additional genomic information that is classified according to the degree of clinical actionability and age of onset or the age at which intervention would be initiated. The study examines the efficacy of an interactive, web-based decision aid intended to support parental informed decision-making by providing education about genomic sequencing and the kinds of conditions that could be identified. The decision aid is designed to help parents explore the degree to which having NGS-NBS is either congruent or in conflict with their values and preferences and precedes an in-person consent visit by a genetic counselor [20]. Parents randomized to the decision arm also view a second web-based decision aid designed to support informed decision-making about three categories of additional information they can learn from their child’s or infant’s genome. Questionnaires gather data to evaluate correlates and processes of parents’ decision-making and their decision outcomes, as well as the psychosocial impact of screening. Additional analyses will explore moderators of effects of learning about and being able to request additional information from an infant’s or child’s sequencing, secondary outcomes, and couples’ decision processes. The study consists of three phases, which are summarized in the study design workflow (Fig. 1) and the SPIRIT figure (Fig. 2).

### Eligible participants and recruitment

Participants can be either sex and of any race or ethnicity and must be fluent in English or Spanish. Parents and their eligible child are enrolled from two cohorts: the well-child cohort or the diagnosed cohort. The well-child cohort is recruited prenatally and consists of parents with a normal intrauterine pregnancy of 18 weeks or greater with no pending or positive prenatal diagnostic test results for congenital malformations or chromosomal abnormalities (see Table 1 for additional inclusion and exclusion criteria). The diagnosed cohort consists of parents and children up to age 5 who have disorders identified through current NBS in North Carolina. These include metabolic disorders (such as phenylketonuria and MCADD), cystic fibrosis, CFTR-related metabolic syndrome, congenital hearing loss, primary ciliary dyskinesia, and other rare conditions (including some that have been recommended for inclusion on the NBS panel, but for which no screening method exists).

**Table 1:** Inclusion and exclusion criteria

	Both cohorts	Well-child cohort	Diagnosed cohort
Inclusion criteria	Parents (couple) or mothers: • At least 18 years old • For mothers who are married or in a marriage-like relationship, their partners must also consent to participate. Mothers who are not married or in a marriage-like relationship will be able to participate individually • Must be able to provide informed consent for their child and for themselves • Must be fluent in English or Spanish	Parents (couple) or mothers: • Pregnant with an intrauterine pregnancy of 18 weeks or longer • Have been identified by medical personnel in the obstetric clinic as eligible candidates. Newborns: • Have no pending or positive prenatal diagnostic test results for congenital malformations or chromosomal abnormalities • Have no complications at the time of birth or unexpected medical problems; however, depending on their clinical course, a sample may be obtained from infants whose parents have previously consented to the study once they have stabilized and been discharged from the neonatal intensive care unit, if the parents agree.	Infants and children from 0 to 5 years who are medically stable and • Diagnosed with a known or suspected monogenic disorder, such as: - Phenylketonuria - Medium chain acyl-CoA-dehydrogenase deficiency (MCADD) - Cystic fibrosis or CFTR-related metabolic syndrome - Congenital hearing loss - Other rare disorders such as primary ciliary dyskinesia or mucopolysaccharidosis OR • Had a positive newborn screen but follow-up testing was non-confirmatory (false positive)
Exclusion criteria	Parents (couple) and mothers: • Younger than 18 years old • Unwilling to complete study procedures • Have cognitive or other impairments that preclude them from giving informed consent • Disagree about their child’s participation • Transfer their prenatal care to another institution • Are not fluent in English or Spanish Newborns and children: • Do not meet the diagnostic criteria as above • Medically unstable • Medical care transferred to another institution		

Due to the assessment by the institutional review board (IRB) at the University of North Carolina at Chapel Hill (UNC) that the study poses more than minimal risk to the child, both members of a couple are required to provide signed informed consent for testing. Therefore, both are recruited to the study except if fathers are determined not to be reasonably available [21]. In those cases, mothers are eligible to participate independently. Otherwise, both parents are expected to attend the consent visit. One parent can consent by telephone in the rare cases where it is not possible for both parents to attend.

Potential participants in the well-child cohort are recruited in-person during a regularly scheduled visit at the obstetrics clinic at UNC Hospitals. Interested couples who agree to hear about the study are given a brochure and an information sheet that describes study activities. Parents in the diagnosed cohort are recruited by a letter from their child’s clinician and are also sent the brochure, the information sheet, and an opt-out card. If parents in either cohort consent to join phase 1 of the study, they will complete an online baseline questionnaire (time 1) and use an online decision aid that will help them decide whether to accept NGS-NBS for their child. If they prefer, parents can complete a paper version of the time 1 questionnaire while they are waiting for their appointment. Parents who do not complete the time 1 questionnaire within 2 weeks are mailed a paper version, along with an addressed stamped envelope.

During the initial stage of the study, an age-based semi-quantitative metric was developed based on previous work by this research group [22]. This metric was used to score and categorize the different types of potential genomic findings to guide informed decision-making and the disclosure of results. In addition to considerations of the age of disease onset, the metric uses the concept of medical actionability, which includes the likelihood and severity of disease outcomes and the efficacy and potential harms of interventions. The metric was used to score gene–disease pairs to determine their eligibility to be included in a NGS-NBS panel that includes genes associated with disorders that are currently in the RUSP as well as other conditions with onset in infancy or childhood that have treatment, monitoring, or medical management that can be reasonably expected to improve outcomes (are medically actionable). Because the conditions identified by the NGS-NBS panel are comparable to those detected by current NBS screening, the results of the analyses of these genes are disclosed to all study participants.

### Categories of genomic information

Conditions that did not meet eligibility for the NGS-NBS panel include those that scored low on the medical actionability criteria or had onset in adulthood. Parents randomized to the decision arm are eligible to request variant analysis in three additional categories: (1) adult-onset conditions with high medical actionability scores, in which the onset or initiation of screening protocols occurs after age 18, such as hereditary breast and ovarian cancer; (2) childhood-onset conditions that have no specific medical interventions and thus, scored low on medical actionability, such as Tay–Sachs disease; and (3) carrier status for autosomal recessive disorders, such as cystic fibrosis, that have reproductive, but rarely personal health implications. Variants associated with adult-onset conditions that scored low on medical actionability, such as amyotrophic lateral sclerosis, are neither analyzed nor offered as a choice for parents.

### Sequencing methods

Duplicate saliva samples from the children and infants are collected and labeled with study ID numbers. Raw sequence data are analyzed using standard bioinformatics methods to map sequence fragments and align them to the reference human genome. Genetic variants are identified using a custom pipeline, deposited in a dedicated database, and extensively annotated and subjected to in silico analysis [23, 24]. Clinical interpretation of the variants follows criteria established by the American College of Medical Genetics and Genomics [25]. However, thresholds for reporting variants depend on the cohort and category of results (Table 2).

**Table 2:** Categories of results that are returned to the two cohorts of the NC NEXUS study

Categories of results	Well-child cohort	Diagnosed cohort
NGS-NBS results	Only pathogenic variants	Only pathogenic variants
Diagnostic results	Not applicable	Results of indication-based analysis for diagnosed conditions: phenylketonuria, MCADD, cystic fibrosis, hearing loss, lysosomal storage diseases, adrenoleukodystrophy, primary ciliary dyskinesia Pathogenic, likely pathogenic, and variants of unknown significance reported
Categories of information that parents randomized to the decision arm are eligible to request:		
Childhood-onset non-medically actionable results	Pathogenic and likely pathogenic variants reported	Pathogenic and likely pathogenic variants reported
Adult-onset medically actionable results	Pathogenic and likely pathogenic variants reported	Pathogenic and likely pathogenic variants reported
Carrier results	Pathogenic and likely pathogenic variants reported	Pathogenic and likely pathogenic variants reported

### Genomic analyses

#### NGS-NBS analysis

This type of analysis is carried out for both cohorts. Given the large number of possible genomic findings and the low a priori likelihood that individuals in the well-child cohort would be affected with any given rare genetic disorder, we chose to strike a balance between the sensitivity and the specificity of the analysis. This balance enables detection of individuals at high risk for a treatable genetic condition without overwhelming clinicians and participants with large numbers of variants of uncertain significance. Thus, in the NGS-NBS panel, only variants determined to be known pathogenic or likely pathogenic and consistent with the expected inheritance pattern of the suspected condition are disclosed.

#### Indication-based analysis

In the diagnosed cohort (children with conditions previously identified by NBS), we perform an indication-based analysis that evaluates variants in genes within a specific diagnostic list constructed to interrogate all known genes that could be related to a patient’s phenotype. In the setting of a diagnostic evaluation, all variants in genes that could be related to the phenotype are prioritized computationally and analyzed. Because these children have already been diagnosed with a rare genetic disorder, variants deemed to be pathogenic, likely pathogenic, or a variant of uncertain significance are returned, according to accepted practice guidelines developed by the American College of Medical Genetics and Genomics.

### Intervention and control conditions

After their NGS-NBS findings are ready to be disclosed, parents of children in both cohorts are randomized to one of two study arms: the decision arm or the control arm (Table 2). Parents assigned to the control arm are not eligible to request additional findings from their infant’s or child’s sequencing. Parents assigned to the decision arm are educated about the scope of additional findings, as described in phase 2 below, and can request any or all of the three categories.

### Randomization and concealment

Parents are randomized in a 2:1 allocation ratio to the decision arm or to the control arm, respectively. Computerized randomization uses permuted block randomization with blocks of randomly varying size. Participants are stratified for block randomization based on three parameters: study cohort (diagnosed or well-child), language preference (English or Spanish), and the relationship status of the parent(s) giving consent (single mother or couple). Random assignments are concealed electronically using an automated computer tracking system that does not reveal assignments until the time of disclosure. The genetic counselor who schedules the disclosure visit, who does not collect questionnaire data from participants, logs into the system to initiate a participant’s random assignment. Parents in the decision arm are instructed to complete the second decision aid prior to their visit and are scheduled far enough in advance to give them time to do so. Once participants are scheduled, study staff who manage data collection and participant communication receive an automated email alert that prompts them to send a customized email message to participants. This email includes information and materials relevant to the participant’s assigned condition and a reminder about the date and time of the disclosure visit.

### Decision aid

To help parents make informed decisions about whether to accept genomic sequencing for their child and about their preferences for additional information, we developed a web-based decision aid grounded in principles of informed decision-making. The content and design of the decision aid were shaped by insights learned from the NC NEXUS steering committee, including the extensive clinical experience of several members, user testing, parent interviews, a discrete choice experiment, and standards suggested for decision aids [26–29]. Adopting a user-centered design approach enabled us to create educational materials that are accessible and relevant to parents, without sacrificing scientific accuracy [20]. The multimedia interface allows parents to go at their own pace, repeating and reviewing information as needed. Plain-language text, graphics, and audio narration are used throughout to convey challenging concepts. The content was organized into three broad sections: education, deliberation, and decisions. Much of the education sections focused on defining terms and explaining what decisions we would ask parents to make. This included describing the NC NEXUS study procedures, providing background on NBS and genomic sequencing, and explaining the kind of results associated with the NGS-NBS panel and, for parents in the decision arm, the additional variant analysis. The deliberation portions of the decision aid were a set of interactive tasks designed to engage parents in the decision-making process and to encourage critical thinking about how participating in the study and learning sequencing results fits with what is most important to them. In the last section, parents indicate what they intend to do and are provided with tailored next steps based on those intentions. The decision aid was tailored to match parents’ study cohort (well-child or diagnosed), language preference (English or Spanish), and relationship status (single or married).

### Trial procedures

#### Phase 1: Education and initial interest in NGS-NBS

After joining the study, each parent in both cohorts is sent a personalized link to the time 1 (pre-decision baseline) questionnaire, which is delivered through Qualtrics (Provo, UT, USA) and optimized for use on mobile phones, tablets, and computers. The information that associates these links with personal data is maintained in a secure database. If parents do not complete the time 1 questionnaire within 1 week of receiving the link, study staff responsible for communicating with participants implement a set schedule of email and phone reminders to encourage parents to complete the questionnaires at their earliest convenience. The time 1 questionnaire collects the information summarized in the phase 1 (pre-decision phase) section of Table 3.

**Table 3:** Timing and measures

Measure	Citation	Scale information	Time 1 (pre-decision baseline, phase 1)	Time 2 (post-decision, phase 1 or 2^a^)	Time 2A (post-additional information decision,^b^phase 2)	Time 3 (short-term follow-up)	Time 4 (long-term follow-up)
Sociodemographic variables			X				
Health literacy	[32]	1 item Likert scale	X				
Personal and family history of genetic testing		5 items Yes/no	X				
Knowledge of genomic sequencing	[38]	19 items True/ false	X	X		X	
Understanding NC NEXUS		5 items True/false	X				
General depression and anxiety	[43]	14 items Likert scales	X			X	X
Decisional conflict	[44]	16 items Likert scale		X	X		
Decision regret	[45]	5 items Likert scale				X	X
Decision regret for additional information^c^	Adapted from [45]	6 items Likert scale				X	X
Dyadic decision-making conflict		9 items Likert scale				X	X
Test-related distress	[46]	17 items Likert scale				X	X

^a^The time 2 questionnaire is administered in phase 1 for participants who decline NGS-NBS in the decision aid and in phase 2 for those who indicate interest in NGS-NBS in the decision aid by selecting accept or undecided

^b^This questionnaire is completed by decision arm participants after they decide to learn all, some, or none of the three categories of additional information

^c^ Relevant to decision arm participants only

After both parents complete the questionnaire, each is sent a link to the decision aid that describes the NGS-NBS analysis. Parents use the decision aid to indicate their initial interest in NGS-NBS: (1) decline, (2) accept, or (3) undecided. Parents who choose option 1 complete a time 2 (post-decision) questionnaire and end their study participation. Parents who choose options 2 or 3 are contacted to schedule a study visit (visit 1) with a certified genetic counselor to begin the post-decision phase (phase 2).

#### Phase 2: NGS-NBS decision and consent at visit 1, randomization, and disclosure of NGS-NBS results at visit 2

Parents who selected options 2 or 3 in the decision aid meet with a certified genetic counselor at visit 1. The counselor provides additional information about genomic sequencing, the risks and benefits of testing, and the types of results that can be expected depending upon the cohort. Depending on the parents’ decision about NGS-NBS, the genetic counselor obtains signed parental informed consent for genomic sequencing and the disclosure of NGS-NBS results to all participants and, for those in the diagnosed cohort, the results of the indication-based analysis. Duplicate cheek swab samples are collected from the children in the diagnosed cohort. Samples from the well-child cohort are obtained after the baby’s birth. After the completion of visit 1, each parent is emailed a link to the time 2 (post-decision) questionnaire (Table 3). Parents who are unresponsive to repeated efforts to schedule visit 1 are sent a letter asking if they still wish to have NGS-NBS or have changed their minds. They are also asked if they would be willing to complete the second questionnaire to enable intent-to-treat analyses.

Once NGS-NBS results are ready, participants are scheduled for a disclosure visit (visit 2), and they are randomized to the decision arm or the control arm prior to visit 2. Parents randomized to the decision arm are emailed a link allowing them to access the second decision aid that describes the three categories of additional information that they are eligible to request from their infant’s or child’s sequencing. They complete a values-clarification exercise about this decision and report their intentions to learn any, all, or none of the categories of additional information.

During visit 2, all parents learn the results from their infant’s or child’s NGS-NBS (i.e., findings associated with medically actionable conditions of childhood onset). The parents of children in the diagnosed cohort also learn the results of their child’s indication-based analysis. All disclosed variants are confirmed by Sanger sequencing in the clinical laboratory at UNC Hospitals, which is approved by the Clinical Laboratory Improvement Amendments program, and clinical reports are generated. Reports are approved by a board-certified molecular geneticist or pathologist, and they are eligible to be included in the electronic health record at the parents’ discretion.

For those in the decision arm, the clinicians then discuss the additional categories of information parents can request and ask them to make their decision at that visit. Parents may request results from some, all, or none of the three categories of additional information (Table 2). Immediately after visit 2, parents in the decision arm are emailed their personal link to complete an online post-decision questionnaire about their decision about whether to learn additional information (time 2A questionnaire). This questionnaire is completed by decision arm parents only; it documents their experiences and responses to learning about and making a decision about additional information.

Because there are multiple steps in this study, we anticipate that parents may become unavailable, change their minds about participation, or discontinue participation. If one parent becomes unavailable after visit 1 (e.g., moves to another state), the remaining parent is able to participate as an individual. If we are unable to schedule visit 1, parents are encouraged to complete the post-decision questionnaire as described above.

#### Phase 3: Disclosure of the results of additional information (decision arm only) and follow-up questionnaire (all parents)

During phase 3, parents in the decision arm who have requested results from the carrier status category can learn these results by telephone. If they have requested information from either or both of the other categories (childhood-onset conditions with low or no medical actionability or adult-onset conditions with high medical actionability), they are scheduled for a third in-person study visit (visit 3) for disclosure by a medical geneticist and genetic counselor. All parents, regardless of their randomly assigned study group, complete two additional follow-up questionnaires after visit 2: the time 3 questionnaire, which collects data for a short-term follow-up within 2 weeks [30, 31] of the return of results (visit 2 for those in the control arm and visit 3 for those in the decision arm), and the time 4 questionnaire, which collects data for a long-term follow-up 3 months after the participants’ final visit (Table 3).

#### Data monitoring

Study data flows are coordinated by workflow management software that directs interaction among different software systems that are isolated to maintain the security of different types of study information. Participant demographic data and consent documents are collected and managed using the REDCap [31] electronic data capture tools hosted at UNC. Survey data are obtained using direct entry by parent participants. Participants are provided individualized web links using a secondary coded identifier, so that the primary coded identifier is protected. Questionnaires are provided using the Qualtrics survey tool. Bioinformatics processing of sequence data are performed on systems isolated from the REDCap instance and use only the coded participant identifiers. The project workflow management software communicates with the decision aid software, the Qualtrics survey tool, and the bioinformatics processing software using defined interfaces over transport layer security.

#### Measures

Self-report measures are administered through online questionnaires (Table 3). When participating as a couple, each parent completes the questionnaires independently. We emphasize that we use measures that are valid, reliable, brief, and appropriate for a diverse population. The measures assess variables needed to describe the sample potential confounds that may need to be included as covariates during analyses, and self-reported primary and secondary outcome variables. Participants complete up to five assessments. All participants complete a time 1 (pre-decision baseline) questionnaire, which is sent electronically before they complete the NGS-NBS decision aid, and a time 2 (post-decision) questionnaire. Participants who decline NGS-NBS in the first decision aid complete the time 2 questionnaire after indicating their choice in the decision aid and then their participation ends, whereas those who indicate accept or undecided in the decision aid enter phase 2 and complete the time 2 questionnaire after visit 1, when they make their final decision about NGS-NBS. Participants in the decision arm also complete a time 2A (post-additional information decision) questionnaire after visit 2. In phase 3, all participants complete two more questionnaires. In the control arm, participants complete the time 3 questionnaire (short-term follow-up) after visit 2. In the decision arm, the time 3 questionnaire is completed after visit 3. In both arms, a final questionnaire (long-term follow-up) is completed 4 months after their final visit.

Sociodemographic variables are collected in the time 1 questionnaire. Both parents report individual-level variables, which include age, gender, ethnicity (Hispanic or non-Hispanic), race, marital status, education, employment status, and health insurance status. Mothers report relationship-level demographic characteristics, which include family income, length of relationship, and parity. Both parents’ health literacy is also assessed at time 1 with the Single Item Literacy Screener [32].

Measures assessing parents’ reactions to the study and to decision-making are summarized in Table 3. These measures include the self-reported primary outcome of decision regret and secondary outcomes including test-related distress, decisional conflict, understanding of NC NEXUS study features, general depression, and anxiety.

#### Planned analyses

Analyses will begin with descriptive statistics for all study variables. We will evaluate the distribution of continuous variables and, if necessary, apply normalizing or variance stabilizing transformations before conducting further analyses. We will use current methods for evaluating patterns of missing data and for imputing missing values, if appropriate. Analyses will evaluate the success of randomization (i.e., to ensure that participants in the study arms do not differ on demographic variables or the child’s medical variables) and include any factors shown to differ as covariates in multivariate models. We will also use an intent-to-treat approach to compare groups. The analytic approach for analyses will be determined by the nature of the outcome (discrete or continuous), need to include covariates, and the research question being addressed. Analyses will be performed by an experienced biostatistician and will apply currently recommended approaches used in randomized controlled trials and subgroup analyses [33]. For example, one analysis will focus on one of the three primary participant-reported outcomes: mothers’ decision regret assessed at the time 3 short-term follow-up. We will conduct an analysis of covariance that includes a variable identifying the assigned study arm (decision or control) and any necessary covariates, selected from demographic or medical variables found to differ across the two study arms at the time 1 pre-decision baseline assessment (if any such imbalance is detected) as well as demographic or medical variables associated with the outcome. If learning about and making a decision about additional information from a child’s sequencing increases regret at having decided to join the study, then we would expect this outcome to be elevated in the decision arm. We will also explore the cohort/study arm interaction to evaluate whether the effects of assignment to the decision arm differ across cohorts. Statistical adjustments for multiple comparisons will not be used, given the nature of our planned analyses.

#### Sample size

NC NEXUS will seek to enroll 200 children into the well-child cohort and into the diagnosed cohort (400 children total), along with their parent or parents, with enrollment indicated by consent to participate in phase 1 of the study. Power analyses were conducted to evaluate statistical power for evaluating the effects of randomizing parents to make the decision (or not) to request additional information from their child’s sequencing. Using PASS Version 11 [34], we estimated the statistical power for detecting the difference in mother’s decision regret. Given Cohen’s *d* = 0.3, which is an effect size considered to be clinically meaningful in patient-reported outcome data [35], the study has a statistical power of 81% to detect the difference under type I error alpha = 0.05 and sample size *n* = 400. It also has sufficient power to explore a multiple regression model predicting mothers’ decisional regret with two covariates of substantive interest (cohort and race/ethnicity) and 10 covariates in the model (e.g., demographics) that account for 20% of the variance in scores. We assumed the sample will be split equally across racial/ethnic groups under complete randomization, which is consistent with the distribution in our study population. If study group and race/ethnicity account for 2% or more of the variance after controlling for the other variables, we will have statistical power of at least 82% under alpha = 0.05, indicating acceptable power for comparisons by study group and race/ethnicity.

### Potential harms

The risks of study participation can be divided between those generic risks inherent to genetic research with human subjects and risks that are unique to the NC NEXUS project. Although the potential harms of genetic testing in the newborn period have been hypothesized (vulnerable child syndrome, genetic discrimination, parental bonding, among others), few data are available to document whether these harms occur and their duration and magnitude [36, 37]; however, the use of genome-scale sequencing heightens the need to collect these data. NC NEXUS was specifically designed to gather empirical evidence regarding potential benefits and harms of the use of NGS in NBS. Although longitudinal studies are needed to characterize them fully, this study seeks to identify potential risks associated with parental responses to learning genetic information about their child. To minimize the psychological risks, the disclosure of positive results is done in conjunction with genetic counseling, and referrals to specialists are made as needed. In addition, the clinical team that has contact with study participants is trained to recognize and probe indicators of possible distress (e.g., participants’ description or display of distress-related symptoms). A measure of participant distress (depressive symptoms and anxiety) is included in questionnaires that participants complete before and after the results are disclosed to establish baseline levels and examine changes in distress over time [38]. Finally, the decision aid was designed to help families understand the risks and benefits of study participation and to make an informed decision based on their values and preferences. This information is expanded and personalized during the in-person informed consent visit. As with all research studies, a study participant may discontinue their involvement at any time.

All clinical information is kept confidential, and only accessed by those directly involved in the research. The digital file containing the linked participant names, UNC medical record numbers and unique study identifiers is stored in a password-protected REDCap database managed by the North Carolina Translational and Clinical Sciences Institute. All identifiers (name, date of birth, etc.) are removed from the saliva samples prior to being sent for DNA extraction.

### Ethical and regulatory considerations

The NC NEXUS study has had regulatory oversight by the Food and Drug Administration (FDA) since the beginning of the study. Based on concerns about the potential harms of returning predictive genomic results to healthy newborns, the FDA declared NC NEXUS a significant risk device study (investigational device exemption G150258). The study is approved by the Biomedical IRB of UNC.

The data in this study is reviewed regularly by an independent data monitor as required by investigational device exemption G150258, in accordance with established good clinical practices of the International Conference on Harmonization (E6 5.1.1, 5.2, 5.18.1) and 21 CFR 812.40 [39], and a clinical monitoring plan developed specifically for the NC NEXUS project by the study data monitor. The role of the data monitor is to review study documentation, regulatory files, and informed consent to ensure the quality and integrity of the data collected and adherence to good clinical practices. An important focus of the data monitoring is to ensure appropriate informed consent has been obtained from the research participants. Areas of deficiencies and timelines for resolution will be documented and agreed upon by the study team and the monitor.

Following the FDA Guidelines for the Establishment and Operation of Clinical Trial Data Monitoring Committees Section 2 [40], a formal data monitoring committee is not needed for trials that are not “intended to prolong life or reduce risk of a major adverse health outcome such as a cardiovascular event or recurrence or cancer, … unless the trial population is at elevated risk of more severe outcomes.”

## Discussion

The NC NEXUS study is led by an interdisciplinary team and is examining the technical, clinical, and ethical issues associated with utilizing genome-scale sequencing technology to screen newborns. The benefits of genome-scale diagnostic testing have largely been demonstrated; however, the use of genomic sequencing as a public health screening tool merits systematic study. Expanding the amount and types of information parents can learn about their newborns will fundamentally alter the limited role parents have traditionally played in this setting. NC NEXUS will study not only how parents make decisions about NBS that incorporates NGS, but it will also advance knowledge by using a rigorous randomized controlled trial design to investigate the effects of being given, versus not being given, a choice to learn additional findings from a child’s sequencing.

Though relevant to the ethical application of NGS technologies to genetic screening in any population, the issues stemming from the pediatric setting are particularly controversial because policy recommendations have generally concluded that asymptomatic testing of minors should be done only when timely identification is needed to prevent harm and directly benefit the child. In addition, factors that influence the decision to learn genetic information has largely focused on adults deciding about their own genome; much less is known about surrogate decision-making, as occurs with NBS.

The potential to expand significantly the number and types of conditions identified by NBS underscores the need for studies of the specificity and sensitivity of NGS for detecting disorders with a genetic etiology in asymptomatic individuals. There is a profound lack of data about the predictive value of genomic sequencing in a public health screening setting, and the difficulty of interpreting the pathogenicity of novel and rare genetic variants is compounded by the lack of clinically relevant phenotypes for comparative inference. Enrollment of cohorts with known disorders identified through recent routine NBS will allow us to assess, in a blinded fashion, the sensitivity of our NGS-NBS panel and workflow for three types of conditions most commonly detected through traditional NBS methods.

In addition, determining those genes for which identified pathogenic variants would be medically actionable as well as those that, while not providing direct medical benefit to the child, could benefit the family, is a significant focus of this study. One novelty of the NC NEXUS study is in its examination of how parents in real-world settings make decisions about a wide range of genetic information and the subsequent impact of these decisions. By identifying and disclosing genetic information that extends beyond the original purpose of NBS, the study may help redefine the disclosure criteria in the newborn period.

The complexity and breadth of information obtainable through genome-scale sequencing could disrupt the process by which NBS is currently delivered, which is a setting in which parents are typically not well informed about testing and are not asked to provide consent. If parents are expected to make decisions about learning information that may not meet current thresholds of actionability, they will need specific guidance about how to decide which information they would prefer to learn and which they would not. Integral to this process is the elicitation and incorporation of their beliefs and values into their personal assessments of the benefits and risks of learning various types of information. Existing educational efforts are inadequate to achieve this goal and new, validated tools will need to be developed. The NC NEXUS electronic decision aid was designed with this goal in mind. It combines the theory and principles of informed decision-making to teach parents about the testing and the kinds of results they might expect. It also helps them to predict their reactions to learning this information and to identify their options.

Increasing public awareness about the potential uses of genetic information has driven the expectation that genomic testing modalities will play a major role in diagnosing, treating, and preventing disease in the future. For example, commercial laboratories currently market direct-to-consumer genomic sequencing of newborns [41, 42], heightening concerns about the clinical validity and long-term impact of returning different types of genetic information to parents of newborns. These concerns, coupled with the likelihood that genomic screening technology will eventually be adopted into neonatal public health screening, emphasize the need for a practical and ethical infrastructure, including validated decision-support materials and an evidence-based process, by which to apply genomic sequencing to NBS for the tangible benefit of parents and children.

Genomic sequencing offers great promise for detecting causative mutations in actionable early-onset conditions that are not currently detectable by traditional NBS methods; however, more research is needed to understand the inherent ethical, social, and public health dilemmas. A broader understanding of several aspects is needed to facilitate incorporation of NGS into newborn public health screening and inform development of important future NBS policy guidelines. These include how parents understand NGS-NBS, the decisions that they make, the role of a decision aid and the supporting clinical interactions in influencing parents’ decisions, and the ramifications of disclosure for family adaptation. We are optimistic that the NC NEXUS study will provide highly relevant information to address the central challenges facing the clinical implementation of genome-scale analysis in children and contribute to the establishment of best practices for NBS.

### Trial status

Patient recruitment began on 14 June 2016 under Version 1 of IRB protocol 13–2409 and will end on 1 May 2018.

## Additional file

Additional file 1: SPIRIT 2013 Checklist: Recommended items to address in a clinical trial protocol and related documents. (DOC 255 kb)

## Abbreviations

dbGaP: Database of Genotypes and Phenotypes
FDA: U.S. Food and Drug Administration
IRB: Institutional review board
MCADD: Medium chain acylCoA-dehydrogenase deficiency
NBS: Newborn screening
NC NEXUS: North Carolina Newborn Exome Sequencing for Universal Screening
NGS: Next-generation sequencing
NSIGHT: Newborn Sequencing in Genomic Medicine and Public Health
RUSP: Recommended Uniform Screening Panel
UNC: University of North Carolina at Chapel Hill
